# First-in-human phase 1 study of budigalimab, an anti-PD-1 inhibitor, in patients with non-small cell lung cancer and head and neck squamous cell carcinoma

**DOI:** 10.1007/s00262-021-02973-w

**Published:** 2021-07-03

**Authors:** Antoine Italiano, Philippe A. Cassier, Chia-Chi Lin, Tuomo Alanko, Katriina J. Peltola, Anas Gazzah, Her-Shyong Shiah, Emiliano Calvo, Andrés Cervantes, Desamparados Roda, Diego Tosi, Bo Gao, Michael Millward, Lydia Warburton, Minna Tanner, Stefan Englert, Stacie Lambert, Apurvasena Parikh, Daniel E. Afar, Gregory Vosganian, Victor Moreno

**Affiliations:** 1 Department of Medical Oncology, Institut Bergonié, 229 Cours de l’Argonne, 3300 Bordeaux, France; 2 University of Bordeaux, Bordeaux, France; 3 Department of Medical Oncology, Centre Leon Berard, Lyon, France; 4 Department of Oncology, National Taiwan University Hospital, Taipei, Taiwan; 5 Docrates Cancer Center, Helsinki, Finland; 6 Department of Drug Development (DITEP), Gustave Roussy, Université Paris-Saclay, Villejuif, France; 7 Graduate Institute of Cancer Biology and Drug Discovery, Taipei Medical University Hospital, Taipei, Taiwan; 8 START Madrid-CIOCC, Centro Integral Oncológico Clara Campal, Hospital Madrid Norte-Sanchinarro, Madrid, Spain; 9 Medical Oncology, Biomedical Research Institute INCLIVA, University of Valencia, Valencia, Spain; 10 CIBERONC, Instituto de Salud Carlos III, Madrid, Spain; 11 Medical Oncology Department, Institut du Cancer de Montpellier, Montpellier, France; 12 Blacktown and Westmead Hospitals, Sydney, NSW Australia; 13 Linear Clinical Research, University of Western Australia, Nedlands, WA Australia; 14 Department of Oncology, Tampere University Hospital, Tampere, Finland; 15 AbbVie Deutschland, GmbH & Co KG, Ludwigshafen, Germany; 16 AbbVie Inc, Redwood City, CA USA; 17 START Madrid-FJD, Hospital Universitario Fundacion Jimenez Diaz, Madrid, Spain; 18 Comprehensive Cancer Center, Helsinki University Central Hospital, Helsinki, Finland

**Keywords:** Budigalimab, Head and neck squamous cell cancer, Non-small cell lung cancer, PD-1 inhibitor

## Abstract

**Background:**

Budigalimab is a humanized, recombinant immunoglobulin G1 monoclonal antibody targeting programmed cell death protein 1 (PD-1). We present the safety, efficacy, pharmacokinetic (PK), and pharmacodynamic data from patients enrolled in the head and neck squamous cell carcinoma (HNSCC) and non-small cell lung cancer (NSCLC) expansion cohorts of the phase 1 first-in-human study of budigalimab monotherapy (NCT03000257; registered 15 December 2016).

**Patients and methods:**

Patients with recurrent/metastatic HNSCC or locally advanced/metastatic NSCLC naive to PD-1/PD-1-ligand inhibitors were enrolled; patients were not selected on the basis of oncogene driver mutations or PD-L1 status. Budigalimab was administered at 250 mg intravenously Q2W or 500 mg intravenously Q4W until disease progression/unacceptable toxicity. The primary endpoints were safety and PK; the secondary endpoint was efficacy. Exploratory endpoints included biomarker assessments.

**Results:**

In total, 81 patients were enrolled (HNSCC: *N* = 41 [PD-L1 positive: *n* = 19]; NSCLC: *N* = 40 [PD-L1 positive: *n* = 16]); median treatment duration was 72 days (range, 1–617) and 71 days (range, 1–490) for the HNSCC and NSCLC cohorts, respectively. The most frequent grade ≥ 3 treatment-emergent adverse event was anemia (HNSCC: *n* = 9, 22%; NSCLC: *n* = 5, 13%). Both dosing regimens had comparable drug exposure and increased interferon gamma-induced chemokines, monokine induced by gamma interferon, and interferon-gamma-inducible protein 10. Objective response rates were 13% (90% CI, 5.1–24.5) in the HNSCC cohort and 19% (90% CI, 9.2–32.6) in the NSCLC cohort. Median progression-free survival was 3.6 months (95% CI, 1.7–4.7) and 1.9 months (95% CI, 1.7–3.7) in the HNSCC and NSCLC cohorts.

**Conclusions:**

The safety, efficacy and biomarker profiles of budigalimab are similar to other PD-1 inhibitors. Development of budigalimab in combination with novel anticancer agents is ongoing.

**Supplementary Information:**

The online version contains supplementary material available at 10.1007/s00262-021-02973-w.

## Introduction

Programmed cell death protein 1 (PD-1), a cell surface protein predominantly expressed on activated T cells, is an inhibitory immune checkpoint receptor and important target for cancer therapy [1, 2]. Its ligands, PD-L1 and PD-L2, are expressed on antigen-presenting cells of the immune system and upregulated in various cancers [3, 4]. Dysregulation of the PD-L1/PD-1 pathway is a mechanism by which malignant cells within the tumor microenvironment subvert protective antitumor immune responses by the host [5, 6], and PD-1/PD-L1 blockade is a promising anticancer strategy. PD-1 inhibitors, such as nivolumab and pembrolizumab, have been evaluated in a number of cancer types, and several PD-1 inhibitors are now approved as monotherapy and in combination with other anticancer agents in multiple cancers, including head and neck squamous cell carcinoma (HNSCC) and non-small cell lung cancer (NSCLC) [7–9].

Budigalimab, formerly called ABBV-181, is a PD-1 inhibitor currently under development. Unlike nivolumab and pembrolizumab, which are both of the immunoglobulin (Ig)G4 subclass, budigalimab is a humanized, recombinant IgG1 anti-PD-1 monoclonal antibody. It has been modified by point mutations (*L*234*A*, *L*235*A*) to reduce Fc receptor interactions and limit effector function. Preclinical experiments have demonstrated that budigalimab exhibits potent PD-1–blocking activity with high specificity [10] and has an affinity similar to that of nivolumab [11] and pembrolizumab [12]. Dose-finding and preliminary safety data from this first-in-human phase 1 study of budigalimab in patients with solid tumors (NCT03000257) have been previously presented [13]. The recommended phase 2 dose was determined to be 250 mg every 2 weeks (Q2W), 375 mg Q3W, or 500 mg Q4W, on the basis of pharmacokinetic (PK) modeling and simulations and PK/pharmacodynamic (PD) assessments that indicated these dosing regimens would lead to comparable exposure ranges and produce similar PD activity and a consistent toxicity profile [14, 15].

This report describes safety, efficacy, biomarker, and PK data from the budigalimab monotherapy expansion HNSCC and NSCLC cohorts of study NCT03000257.

## Patients and methods

### Patient and public involvement

There was no patient or public involvement in design, planned recruitment, or planned dissemination of this study.

### Patient eligibility

Eligible patients were at least 18 years old with advanced HNSCC (arising from the oral cavity, oropharynx, hypopharynx, or larynx) or squamous or nonsquamous NSCLC, Eastern Cooperative Oncology Group (ECOG) performance status of 2 or lower, and measurable disease by Response Evaluation Criteria In Solid Tumors (RECIST; version [v]1.1 [16]. Patients were also required to have adequate organ function (including absolute neutrophil count ≥ 1,500/mm^3^, platelets ≥ 100,000/mm^3^, hemoglobin ≥ 9.0 g/dL, and creatinine clearance ≥ 50 mL/min as assessed by the Cockcroft-Gault formula or 24-h creatinine clearance). Eligible patients in the NSCLC expansion cohort had locally advanced or metastatic NSCLC, had previously experienced platinum-based therapy failure, and were naive to PD-1/PD-L1-targeting agents; in the HNSCC expansion cohort, patients had recurrent or metastatic disease that was not amenable to curative treatment with local or systemic therapy and were naive to PD-1/PD-L1-targeting agents. For this first-in-human study, patients were not selected on the basis of the presence or absence of any particular driver oncogenic mutations nor on their PD-L1 status. Key exclusion criteria included a history of inflammatory bowel disease, immune-mediated pneumonitis, active autoimmune disease (with exceptions of vitiligo, type I diabetes mellitus, hypothyroidism, and psoriasis), primary immunodeficiency, bone marrow or solid organ transplantation, HIV-positive ( +) status, chronic active hepatitis B or C infection, uncontrolled central nervous system metastasis, or evidence of hemolysis on screening laboratory studies.

The study protocol and informed consent form were approved by the institutional review board at each participating site prior to initiation of any screening or study-specific procedures. Written informed consent was obtained from each individual participating in the study. The study was conducted in accordance with the Declaration of Helsinki and Good Clinical Practice guidelines, as defined by the International Conference on Harmonization. This study is registered at ClinicalTrials.gov (NCT03000257).

### Study design and treatment

This was a multicenter, open-label, phase 1 study of budigalimab in adult patients with advanced solid tumors, consisting of two parts: dose escalation and dose expansion. The primary objectives were to examine the safety and PK of budigalimab monotherapy. The secondary objective was to evaluate preliminary activity of budigalimab, and exploratory objectives included (1) evaluation of PD and exploratory biomarkers for association with safety, PK, and clinical responses; and (2) evaluation of baseline PD-L1 expression and relationship with outcome.

The overall study schema is shown in supplementary Fig. 1. The dose-escalation portion of the study followed a standard 3 + 3 design to determine the safety, maximum tolerated dose, and PK profile of budigalimab. On the basis of previously reported safety, PK, and PD data from the dose-escalation portion of the study [13, 14], patients were then enrolled into two tumor-specific monotherapy dose-expansion cohorts, HNSCC and NSCLC, which are reported in this current analysis. Budigalimab was administered by intravenous infusion at either 250 mg Q2W or 500 mg Q4W until disease progression per RECIST v1.1 [16], confirmed disease progression per immune (i)RECIST [17], unacceptable toxicity, or other protocol-defined discontinuation criteria (supplementary Table 1). Patients experiencing radiographic progression per RECIST v1.1 could continue budigalimab treatment if they had no symptoms or signs of disease progression, no decline in ECOG performance status, and no evidence of rapid disease progression or progressive tumor at critical anatomic sites.

Budigalimab was administered as follows: the first infusion was delivered over 90 min; if the patient did not experience an infusion reaction, the second infusion was shortened to 60 min. Subsequent infusions could be administered over 30 min in the absence of infusion reactions following the first or second infusion. Dose reduction of budigalimab was not permitted.

### Assessments

Safety evaluations were performed throughout the study and included assessment of treatment-emergent adverse events (TEAEs) and monitoring of additional clinical data (including vital signs, physical examination, electrocardiograms, echocardiograms, and laboratory test assessments). AEs were graded according to the National Cancer Institute Common Terminology Criteria for Adverse Events v4.03. The criteria for permanent discontinuation of budigalimab following a TEAE are described in supplementary Table 1. Immune-related AEs were managed per published guidelines [18–20].

Intensive serial blood samples for measurement of budigalimab concentrations (PK) in serum were collected in cycles 1 and 3, and additional samples were collected during cycle 2 and cycles ≥ 4. PK parameters were estimated using noncompartmental analysis in Phoenix® WinNonlin® (Certara, Princeton, NJ) and included maximum observed concentration (*C*_max_), time to *C*_max_, area under the concentration–time curve, and half-life.

Biologic samples were collected from each patient to evaluate tumor-specific and systemic biomarkers. All patients consented to provide either archived formalin-fixed paraffin-embedded tumor tissue or a pretreatment, fresh tumor biopsy. Tumor tissue was analyzed for PD-L1 expression using the Dako 28–8 pharmDX IHC [immunohistochemistry] assay (Agilent Technologies, Santa Clara, CA); testing was performed at a single laboratory (Mosaic Laboratories, Lake Forest, CA). Blood samples for exploratory biomarker assessment were collected prior to infusion (0 h, predose), 2-h postinfusion, and on days 2, 3, 8, and 15 in cycles 1 and 3, and days 1 and 15 of cycle 2. Biomarkers evaluated included immune cell counts and PD-1 saturation on CD4 + central memory T cells by real-time flow cytometry, as well as soluble cytokine quantification in cryopreserved serum by Luminex® (Austin, TX).

Efficacy endpoints included objective response rate (ORR; defined as confirmed complete response [CR] or confirmed partial response [PR]), best overall response (CR, PR, or stable disease [SD]), progression-free survival (PFS), and duration of objective response (DOR). Tumor assessments by radiographic imaging (contrast-enhanced computed tomography or magnetic resonance imaging) were performed at baseline and repeated every two treatment cycles for the first 12 months and every three cycles thereafter; these were investigator assessed according to RECIST v1.1 and iRECIST.

### Statistical analyses

Approximately 40 patients were enrolled in each of the HNSCC and NSCLC expansion cohorts to evaluate safety and tolerability of budigalimab. All patients who received any amount of budigalimab were included in the demographic, baseline, and safety analyses. All patients who received at least one dose of study drug and had at least one postdose tumor assessment were included in the efficacy analyses. The two-sided 90% CIs for ORR were provided on the basis of the Clopper–Pearson (exact) method. PFS was defined as time from first dose of study drug to radiographic progression or death, whichever occurred first. For each responder, DOR was defined as time from initial response to the study drug to radiographic progression or death. Both PFS and DOR were summarized using the Kaplan–Meier method.

## Results

### Patient demographics and baseline characteristics

Between November 2017 and January 2019, 81 patients were enrolled in the HNSCC (*N* = 41) and NSCLC (*N *= 40) expansion cohorts (data cutoff: October 31, 2019). For the HNSCC cohort, the first patient was screened on 4 January 2018, and the last patient on 22 January 2019; for the NSCLC cohort, the first patient was screened on 8 November 2017, and the last patient on 20 December 2018. Baseline demographics and clinical characteristics of both cohorts are summarized in Table 1. Sufficient tumor samples for IHC analysis were obtained from 38 patients with HNSCC and 33 patients with NSCLC; 19 patients in the HNSCC cohort and 16 in the NSCLC cohort were PD-L1 + . There was insufficient tumor tissue for analysis from three patients considered responders per RECIST v1.1: 1 patient with HNSCC, and two patients with NSCLC.

**Table 1:** Patient demographics and clinical characteristic

Characteristic, *n* (%)	HNSCC(*N* = 41)	NSCLC(*N* = 40)
Median age, years (range)	62 (51–84)	65 (39–79)
Age		
< 65 years	26 (63)	15 (37)
≥ 65 years	20 (50)	20 (50)
Gender		
Male	35 (85)	23 (58)
Female	6 (15)	17 (43)
ECOG performance status		
0	6 (15)	19 (48)
1	34 (83)	20 (50)
2	1 (2.4)	1 (2.5)
Prior systemic therapies		
1	12 (29)	21 (53)
2	14 (34)	10 (25)
≥ 3	15 (37)	9 (23)^a^
Any prior therapies, *n* (%)	41 (100)	40(100)
*Platinum-containing regimen*		
Cisplatin	32 (78)	18(456)
Carboplatin	23 (56)	17(43)
Cisplatin/Docetaxel/Fluorouracil	2 (5)	0
Carboplatin/Fluorouracil	2 (5)	0
*Targeted therapy*		
Cetuximab	26(63)	0
Erlotinib	0	3(8)
Gefitinib	0	2(5)
Sunitinib	0	1(3)
Afatinib	0	1(3)
EGF816	0	1(3)
Monalizumab	1(2)	0
Osimertinib	0	1(3)
*Bevacizumab-containing regimen*		
Bevacizumab	0	6(15)
*Pemetrexed-containing regimen*		
Pemetrexed	0	20(50)
Histologic type		
Adenocarcinoma	0	30(75)
Neuroendocrine	0	1(3)
Sarcomatoid carcinoma	0	1(3)
Squamous cell carcinoma	41(100)	8(20)
PD-L1 status		
Positive/total tested	19/38 (50)	16/33 (48)
Mutation status (reported or detected positive)^b^		
*EGFR*^c^	–	7
*KRAS*^d^	*–*	7
*ALK* rearrangement^e^	–	1
Budigalimab dosing frequency		
Q2W	31 (76)	19 (48)
Q4W	10 (24)	21 (53)^a^

^a^Percentage > 100 due to rounding. ^b^Mutation status was not collected for HNSCC cohort; for NSCLC cohort, mutation testing was not performed on all patients, but collected if status was known by the investigator. Ten NSCLC patients had sufficient submitted tissue for sponsor to test, resulting in the detection of 1 additional *EGFR* mutation and 1 additional *KRAS* mutation. ^c^One patient with *EGFR* mutation was also PD-L1 + . ^d^Four patients with *KRAS* mutation were also PD-L1 + . ^e^One patient with *ALK* rearrangement was PD-L1 +

+ , positive; *ALK* anaplastic lymphoma kinase; *CNS* central nervous system; *ECOG* Eastern Cooperative Oncology Group; *EGFR* epidermal growth factor receptor; *HNSCC* head and neck squamous cell carcinoma; *NSCLC* non-small cell lung cancer; *PD-L*1 programmed cell death protein 1 ligand 1; Q, every; W, weeks

### Patient disposition and safety

The median duration of exposure to budigalimab was 72 days (range, 1–617) for the HNSCC cohort and 71 days (range, 1–490) for the NSCLC cohort (supplementary Table 2). In total, 24% of patients (*N* = 10) and 33% of patients (*N* = 13) in the HNSCC and NSCLC cohorts, respectively, reported budigalimab dose interruption. As of the data cutoff, two patients in the HNSCC cohort and four patients in the NSCLC cohort continued to receive budigalimab; the reasons for budigalimab treatment discontinuation were progressive disease (HNSCC: 88%; NSCLC: 70%), AEs (HNSCC: 7%; NSCLC: 18%), and withdrawn consent (NSCLC: 2.5%).

All patients (100%) in the HNSCC (*N* = 41) and NSCLC (*N* = 40) expansion cohorts experienced ≥ 1 TEAE. In total, 25 patients (61%) in the HNSCC cohort and 27 patients (68%) in the NSCLC cohort reported grade ≥ 3 TEAEs; the most frequently reported was anemia (HNSCC: *n* = 9, 22%; NSCLC: *n* = 5, 13%). Patients were evaluated for the presence of hemolysis as a cause of anemia; no patients had this condition. TEAEs occurring in ≥ 20% of patients and the most common grade ≥ 3 TEAEs summarized by dose are provided in Table 2.

**Table 2:** Summary of any-grade TEAEs occurring in ≥ 20% of patients and the most frequent (≥ 10%) grade ≥ 3 TEAEs by dose

By MedDRA preferred term, *n* (%)	HNSCC *N* = 41		NSCLC *N* = 40	
	250 mg Q2W(*n* = 31)	500 mg Q4W(*n* = 10)	250 mg Q2W(*n* = 19)	500 mg Q4W(*n* = 21)
Any TEAE	31 (100)	10 (100)	19 (100)	21 (100)
Anemia	8 (26)	2 (20)	7 (37)	4 (19)
Asthenia	14 (45)	2 (20)	5 (26)	0
Constipation	8 (26)	2 (20)	3 (16)	3 (14)
Decreased appetite	9 (29)	1 (10)	2 (11)	3 (14)
Dyspnea	3 (10)	3 (30)	3 (16)	4 (19)
Fatigue	4 (13)	1 (10)	4 (21)	9 (43)
Hypothyroidism	6 (19)	3 (30)	1 (5)	5 (24)
Malignant neoplasm progression	3 (10)	1 (10)	5 (26)	4 (19)
Nausea	8 (26)	1 (10)	1 (5)	2 (10)
Pneumonia	1 (3)	2 (20)	1 (5)	1 (5)
Pruritus	7 (23)	0	3 (16)	0
Grade ≥ 3 TEAE	19 (61)	6 (60)	12 (63)	15 (71)
Anemia	8 (26)	1 (10)	4 (21)	1 (5)
Decreased appetite	3 (10)	0	0	0
Fatigue	3 (10)	0	0	0
Hypercalcemia	3 (10)	0	1 (5)	1 (5)
Malignant neoplasm progression	3 (10)	1 (10)	5 (26)	4 (19)
Acute kidney injury	3 (10)	0	1 (5)	0
Cardiac arrest	0	1 (10)	0	0
Dysphagia	1 (3)	1 (10)	0	0
Mouth hemorrhage	0	1 (10)	0	0
Neck abscess	0	1 (10)	0	0
Cellulitis	0	1 (10)	0	0
Lung infection	1 (3)	1 (10)	1 (5)	0
Pneumonia	0	1 (10)	1 (5)	0
Upper respiratory tract infection	0	1 (10)	0	2 (10)
Tracheal obstruction	0	1 (10)	0	0
Hyponatremia	1 (3)	0	1 (5)	3 (14)
Tumor pain	0	1 (10)	0	0
Dyspnea	0	1 (10)	1 (5)	1 (5)

HNSCC, head and neck squamous cell carcinoma; MedDRA, Medical Dictionary for Regulatory Activities; NSCLC, non-small cell lung cancer; TEAE, treatment-emergent adverse event; Q, every; W, weeks

A total of 26 patients (63%) in the HNSCC cohort and 23 patients (58%) in the NSCLC cohort experienced an AE considered related to budigalimab by investigator assessment; the most common were hypothyroidism (*n* = 8; 20%), diarrhea (*n* = 6; 15%), and pruritus (*n* = 6; 15%) in the HNSCC cohort, and hypothyroidism (*n* = 6; 15%) and fatigue (*n* = 5; 13%) in the NSCLC cohort. Any-grade treatment-related AEs (TRAEs) that occurred in ≥ 10% of patients are summarized in supplementary Table 3. Four patients (10%) in the HNSCC cohort and five patients (13%) in the NSCLC cohort experienced a grade ≥ 3 AE related to budigalimab, with acute kidney injury (*n* = 2; 5%), anemia, diarrhea, and hypokalemia (*n* = 1; 2% each) in the HNSCC cohort, and reduced visual acuity, microscopic colitis, immune-mediated hepatitis, increased transaminase, hyponatremia, and hypophosphatemia (*n* = 1; 2% each) in the NSCLC cohort. Four patients (10%) in the HNSCC cohort and two patients (5%) in the NSCLC cohort experienced a serious TRAE, with acute kidney injury (*n* = 2; 5%), diarrhea, general physical health deterioration, and pyrexia (*n* = 1; 5%) in the HNSCC cohort, and immune-mediated hepatitis and acute kidney injury (*n* = 1; 3% each) in the NSCLC cohort.

The most common TEAEs considered immune-mediated reactions by the investigator, shown in supplementary Table 4, were hypothyroidism (*n* = 7; 17%), diarrhea (*n* = 5; 12%), and pruritus (*n* = 3; 7%) in the HNSCC cohort, and hypothyroidism (*n* = 6; 15%) and maculopapular rash (*n* = 3; 8%) in the NSCLC cohort. Overall, 17 (21%) patients experienced a TEAE that led to study drug discontinuation: 7 (17%) in the HNSCC cohort, and 10 (25%) in the NSCLC cohort (see supplementary Table 5). A single patient in the NSCLC cohort experienced grade ≥ 3 TRAEs leading to budigalimab discontinuation (immune-mediated hepatitis, grade 4). TEAEs leading to budigalimab dose interruption were reported by 14 patients (34%) in the HNSCC cohort and 15 patients (38%) in the NSCLC cohort (supplementary Table 6). The most common TEAEs leading to budigalimab dose interruption were acute kidney injury and dyspnea (*n* = 2; 5% each) in the HNSCC cohort, and upper respiratory tract infection and hypercalcemia (*n* = 2; 5% each) in the NSCLC cohort. No patients experienced a TRAE leading to death during the study; all TEAEs leading to death were considered unrelated to budigalimab (see supplementary Table 7). A single event of grade 5 cardiac arrest occurred. The patient was an 85-year-old male with an extensive history of cigarette smoking and left ventricular hypertrophy. The patient, who had no antecedent history of increasing dyspnea, chest pain, or any immune-related reactions to therapy, died in his sleep. The most likely causes for this event were coronary thrombosis, cardiac arrhythmia, or pulmonary embolism; myocarditis was not considered a likely cause.

### Pharmacokinetics

Budigalimab PK results from dose-escalation and dose-expansion cohorts, across varying doses and regimens, have been reported previously [14, 15]. Budigalimab PK was approximately dose-proportional across the clinical doses evaluated. The two dosing regimens of 250 mg Q2W and 500 mg Q4W resulted in comparable dose-normalized exposures (supplementary Table 8) and maintained receptor saturation, as was previously predicted from population PK modeling and simulations and PK/PD assessments [14, 15].

### Biomarkers

**Fig. 1:**
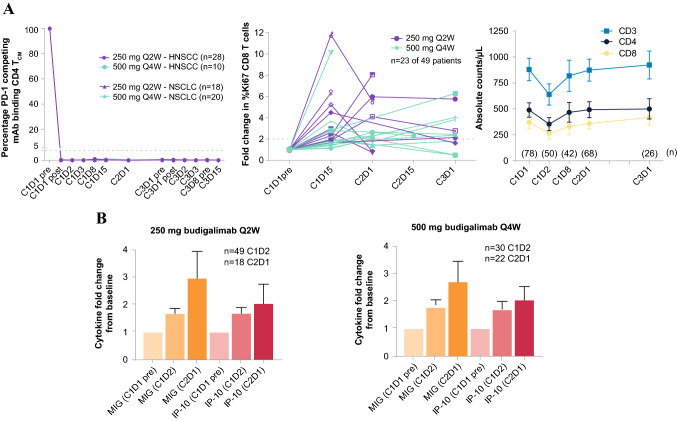
PD-1 receptor saturation and pharmacodynamic effects of budigalimab administration by dose level. Data shown for each patient with assay baseline and at least 1 postbaseline value. Individual patient data shown for patients with ≥ twofold change in CD8 Ki67 staining. Mean + /– 95% CI shown for PD-1 staining, T-cell counts, and cytokines (number of patients for each mean shown in graph). C, cycle; D, day; HNSCC, head and neck squamous cell carcinoma; hr, hour; IP-10, interferon gamma-induced protein 10; mAb, monoclonal antibody; MIG, monokine induced by gamma interferon; NSCLC, non-small cell lung cancer; PD-1, programmed cell death protein 1; Q, every; T_CM_, central memory T cells; W, weeks

Budigalimab demonstrated complete sustained receptor saturation on circulating CD4 + central memory T cells and the expected PD effects at both the 250-mg Q2W and 500-mg Q4W doses (Fig. 1). Complete PD-1 saturation was observed within 2 h of dosing, followed by a transient drop in the number of circulating T cells at cycle (C)1 day (D)2, and increased proliferation of CD8 + T cells in 23 of 49 tested patients (47%), as measured by a ≥ twofold change in Ki67 from baseline (Fig. 1a). Increases in interferon gamma-induced chemokines, monokine induced by gamma interferon (MIG), and interferon gamma-induced protein 10 (IP-10) were observed within a day of dosing and increased through C2D1, with similar kinetics and magnitude of induction observed at 250-mg Q2W and 500-mg Q4W doses (Fig. 1b).

### Antitumor activity

A total of 77 patients were included in the efficacy-evaluable population (HNSCC: *n* = 40; NSCLC: *n* = 37). One patient in the HNSCC cohort discontinued budigalimab prior to week 8 secondary to grade 5 acute respiratory distress syndrome (unrelated to budigalimab), and three patients in the NSCLC cohort discontinued budigalimab (two secondary to clinical progression and one secondary to grade 5 upper respiratory infection, both unrelated to budigalimab).

**Fig. 2:**
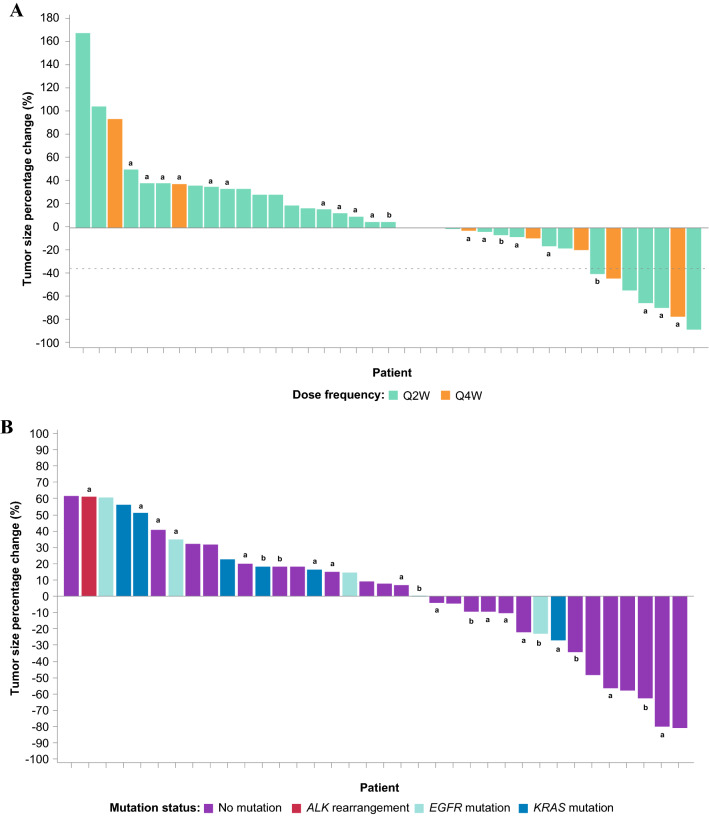
Best percentage change in target lesions from baseline for **a** HNSCC and **b** NSCLC cohorts receiving budigalimab monotherapy. ^a^PD-L1 + status; ^b^PD-L1 status missing (i.e., unknown). ALK, anaplastic lymphoma kinase; EGFR, epidermal growth factor receptor; HNSCC, head and neck squamous cell carcinoma; NSCLC, non-small cell lung cancer; PD-L1 + , programmed cell death protein 1 ligand 1 positive; Q, every; W, weeks

**Fig. 3:**
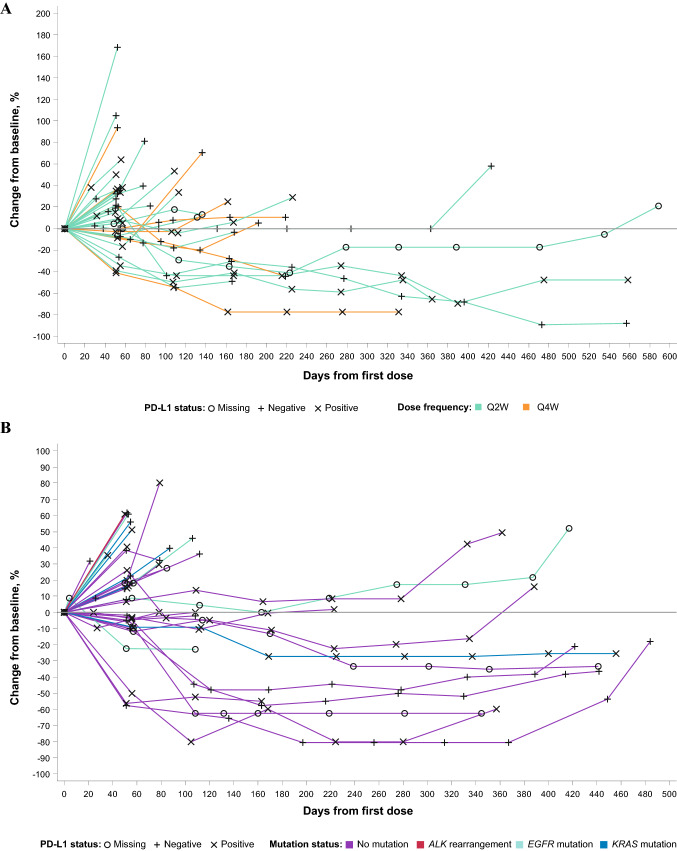
Percentage change in target lesions from baseline for **a** HNSCC and **b** NSCLC cohorts receiving budigalimab monotherapy. ALK, anaplastic lymphoma kinase; EGFR, epidermal growth factor receptor; HNSCC, head and neck squamous cell carcinoma; NSCLC, non-small cell lung V; PD-1, programmed cell death protein 1; Q, every; W, weeks

The best percentage change from baseline in size of target lesions for HNSCC patients is shown in Fig. 2a and for NSCLC patients in Fig. 2b. The percentage change over time in the size of target lesions is shown in Fig. 3a and Fig. 3b for HNSCC and NSCLC patients, respectively. A best overall response (defined as unconfirmed responses as per RECIST v1.1. or iRECIST) of PR or CR was achieved in 15% (90% CI, 6.7–27.5) of patients in the HNSCC and 19% (90% CI, 9.2–32.6) of patients in the NSCLC cohort. The ORR (defined as confirmed responses per RECIST v1.1. or iRECIST) for the HNSCC and NSCLC cohorts was 13% (90% CI, 5.1–24.5) and 19% (90% CI, 9.2–32.6), respectively (Table 3). The ORR for PD-L1 + (≥ 1%) patients in the HNSCC and NSCLC cohorts was 16% (90% CI, 4.5–35.9; 3 confirmed PRs in 19 PD-L1 + HNSCC patients) and 13% (90% CI, 2.3–34.4; 2 confirmed PRs in 16 PD-L1 + NSCLC patients), respectively (Table 3). The ORR for patients with NSCLC who had ≥ 50% PD-L1 expression was 29% (2 of 7 evaluable patients); when patients with epidermal growth factor receptor (*EGFR*) mutation or anaplastic lymphoma kinase (*ALK*) rearrangement are excluded from this group, the ORR was 40% (2 of 5 evaluable patients). Median PFS in HNSCC patients was 3.6 months (95% CI, 1.7–4.7 and 1.9 months (95% CI, 1.7–3.7) in NSCLC patients (Table 3; supplementary Fig. 2); median DOR was 9.4 months (95% CI, 1.9–not estimable) and 10.1 months (95% CI, 7.8–13.1) in the HNSCC and NSCLC cohorts, respectively (Table 3). The Kaplan–Meier estimate for the 6-month DOR rate was 80% in HNSCC and 100% in NSCLC, with only one responder exhibiting progressive disease within 6 months of response. Overall, responses were observed in both PD-L1 + and PD-L1–negative (PD-L1–) patients and were durable. In the NSCLC cohort, no responses were observed in patients with known *EGFR* mutation (*n* = 7), *KRAS* mutation (*n* = 7), or with *ALK* rearrangement (*n* = 1), regardless of PD-L1 expression.

**Table 3:** Summary of best overall response in patients

	HNSCC(*N* = 40^a^)	NSCLC(*N* = 37^b^)
Best overall response [CR + PR], *n* (%) per RECIST v1.1 and iRECIST	6^c,d^ (15)	7 (19)
[90% CI]	[6.7–27.5]	[9.2–32.6]
CR	0	1 (3)
PR	6^c^ (15)	6 (16)
SD	17 (43)	13 (35)
Objective response rate [CR + PR], *n* (%) per RECIST v1.1 and iRECIST	5^c,d^ (13)	7 (19)
[90% CI]	[5.1–24.5]	[9.2–32.6]
Confirmed CR	0	1 (3)
Confirmed PR	5^c^ (13)	6 (16)
PD-L1 + [≥ 1%], *n* (%)	19 (48)	16 (43)
Objective response rate [CR + PR], *n* (%) per RECIST v1.1 and iRECIST	3 (16)	2 (13)
[90% CI]	[4.5–35.9]	[2.3–34.4]
Confirmed CR	0	0
Confirmed PR	3 (16)	2^e^ (13)
PD-L1 + [> 50%], *n* (%)	7 (18)	7 (19)
Objective response rate [CR + PR], *n* (%) per RECIST v1.1 and iRECIST	1 (14)	2 (29)
[90% CI]	[0.7–52.1]	[5.3–65.9]
Confirmed CR	0	0
Confirmed PR	1 (14)	2 (29)
Median DOR, months per RECIST v1.1	9.4	10.1
[95% CI]	[1.9–NE]	[7.8–13.1]
6-mo KM estimate of DOR per RECIST v1.1	0.8	1.0
[95% CI]	[0.20–0.97]	[NE–NE]
Median PFS, months per RECIST v1.1	3.6	1.9
[95% CI]	[1.7–4.7]	[1.7–3.7]
6-mo KM estimate of PFS per RECIST v1.1	0.27	0.27
[95% CI]	[0.14–0.42]	[0.14–0.42]

^a^One patient discontinued budigalimab prior to week 8 secondary to grade 5 acute respiratory distress syndrome unrelated to budigalimab. ^b^Two patients discontinued budigalimab secondary to clinical progression; 1 patient discontinued budigalimab secondary to grade 5 upper respiratory infection, unrelated to budigalimab. ^c^Includes 1 patient meeting criteria for PR per iRECIST. ^d^One patient discontinued budigalimab for disease progression on study day 166 following unconfirmed PR on study day 110. ^e^Both patients with confirmed PR had PD-L1 expression > 50%

*CR* complete response; *DOR* duration of response; *HNSCC* head and neck squamous cell carcinoma; *iRECIST* immune Response Evaluation Criteria In Solid Tumors; *KM* Kaplan–Meier; *NE* not estimable; *NSCLC* non-small cell lung cancer; PD-L1 + , programmed cell death protein 1 ligand 1 positive; *PFS* progression-free survival; *PR* partial response; *RECIST* Response Evaluation Criteria In Solid Tumors; *SD* stable disease; *v* version

## Discussion

This first-in-human phase 1 study demonstrated that budigalimab administration at doses of 250 mg IV Q2W or 500 mg IV Q4W in patients with HNSCC and NSCLC was equally safe and well tolerated. Budigalimab showed dose-proportional PK and had comparable dose-normalized exposures at the evaluated dosing regimens of 250 mg Q2W and 500 mg Q4W.

The safety profile of budigalimab observed in the current study was comparable to that observed with other approved PD-1–targeted agents, including nivolumab and pembrolizumab. The incidents of anemia that was observed following budigalimab treatment was likely due to the prior chemotherapy and radiation therapy that the patients received. Careful monitoring for development of hemolysis during the study found no such events. In the CheckMate 017 study, the most common AEs in patients with advanced-stage squamous NSCLC treated with nivolumab were fatigue, decreased appetite, and asthenia [21]. In CheckMate 057, a study of nivolumab in nonsquamous NSCLC patients, the most common AEs were fatigue, decreased appetite, cough, constipation, and dyspnea [22]. In KEYNOTE-010, fatigue, pruritus, and decreased appetite were the most common AEs reported in NSCLC patients treated with pembrolizumab [23]. Accumulating evidence suggests that only a fraction of cancer patients benefit from immune checkpoint inhibitors, and severe immune-related AEs are associated with immune checkpoint inhibitor therapy [24]. The most commonly reported immune-related AEs reported in the HNSCC and NSCLC cohorts of the present study were hypothyroidism, diarrhea, hyperthyroidism, pruritus, and rash, which are similar to those reported in previous studies of immune checkpoint inhibitors [25].

Antitumor activity was observed following budigalimab treatment, with one patient in the NSCLC cohort achieving CR and six patients achieving PR. In the HNSCC cohort, six patients achieved PR, with one PR per iRECIST criteria after initial progressive disease per RECIST v1.1. Additionally, one patient in the HNSCC cohort and three in the NSCLC cohort achieved immune SD following previous progression per RECIST. The observation of pseudoprogression (disease progression per RECIST followed by subsequent reduction in tumor burden) [26] in several patients enrolled in this study is characteristic of immune checkpoint inhibitors, and similar observations have been reported in studies of other immune checkpoint inhibitors, including ipilimumab, nivolumab, and pembrolizumab [27–30]. Durable responses were observed both in PD-L1 + and PD-L1– patients in the current study, similar to responses observed in clinical studies of other PD-1-targeting agents [21, 22].

Efficacy data from the current study indicate that the clinical activity of budigalimab is similar to that of approved anti-PD-1 agents in patients with NSCLC. Nivolumab exhibited ORRs of 20% [21] and 19% [22] in patients with squamous and nonsquamous NSCLC, respectively, while pembrolizumab exhibited an ORR of 18% in NSCLC patients with tumor proportion score ≥ 1% (KEYNOTE-010) [23]. Of note, most patients treated in these studies had 1 prior line of therapy (99% for CheckMate 017, 88% for CheckMate 057, and 68% for KEYNOTE-010) [21–23]. Such data may indicate that patients with ≥ 2 prior lines of therapy do not derive clinical benefit from these particular checkpoint inhibitors. In the current study, 53% of NSCLC patients treated with budigalimab had received 1 prior line of therapy, while 47% had ≥ 2 prior lines; among the seven responders, six patients had received one prior line of systemic therapy and one responder had received two prior lines of systemic therapy.

Although this study evaluated budigalimab in 40 patients with NSCLC, similar to other anti-PD-1 therapies a lower response rate was observed in NSCLC patients with tumors that harbor *EGFR*-activating mutations and *ALK* rearrangements [31]. The current study exhibited a higher proportion of patients with these genomic alterations (20%), compared with the proportion of patients with *EGFR*-activating mutations and *ALK* rearrangements in the CheckMate 057 (18%) and KEYNOTE-010 (9%) trials [22, 23]. Also, similar to other anti-PD-1 therapies, NSCLC patients treated with budigalimab with high (≥ 50%) tumor PD-L1 expression had higher ORR (29%) compared with the overall NSCLC cohort or with NSCLC patients with confirmed ≥ 1% tumor PD-L1 expression (19% and 13% ORR, respectively) [23]. It is worthy of mentioning that this trial was designed, and patients enrolled, at a time when the key oncogenic drivers in NSCLC were considered to be *EGFR* mutations and *ALK* rearrangements. In the intervening years since trial initiation, a number of other potential driver mutations have been identified in genes such as rearranged during transfection (*RET*), neurotrophic tyrosine receptor kinase (*NTRK*), human epidermal growth factor receptor 2 (*HER2*)*,* and v-raf murine sarcoma viral oncogene homolog B1 (*BRAF*). However, as the majority of patients with NSCLC in this study had insufficient biopsy tissue, we were unable to perform an extended mutational analysis and determine the frequency of these mutations. Further studies may be warranted to evaluate the efficacy of budigalimab in patients with driver mutations other than *EGFR* and *ALK*.

Data from the HNSCC cohort are also consistent with response rates observed for nivolumab and pembrolizumab. In HNSCC, ORRs were 13% for nivolumab [22] and 15% for pembrolizumab (KEYNOTE-010) [23]. Budigalimab demonstrated a 13% ORR, with one responder meeting criteria for immune PR (on study day 101 after meeting criteria for immune unconfirmed progressive disease on study day 51).

PK assessments indicated that the 250-mg Q2W and 500-mg Q4W regimens resulted in similar dose-normalized exposures and PD activity, suggesting that either schedule is viable and thereby providing flexibility in potential combinations with other anticancer agents.

Biomarker assessment of the effect of budigalimab administration on PD-1 receptor occupancy showed complete saturation of PD-1 at 250 mg Q2W and 500 mg Q4W. PD-1 saturation resulted in expected biologic activities on T-cell proliferation and chemokines. These results are consistent with the activity of other anti-PD-1 agents, which enhance antitumor immune activity as detected by increases in peripheral CD8 T-cell proliferation [32, 33], interferon gamma-induced serum chemokines [34, 35], and therapeutic antitumor effects [36].

In conclusion, these data demonstrate that budigalimab has a manageable safety profile with evidence of biologic and clinical activity in patients with previously treated HNSCC and NSCLC that seems to be similar to approved PD-1 inhibitors. The data support the continued development of budigalimab in multiple oncology indications.

### Supplementary Information

Below is the link to the electronic supplementary material.

Supplementary file1 (PDF 727 KB)

## Data Availability

Availability of data and material AbbVie is committed to responsible data sharing regarding the clinical trials we sponsor. This includes access to anonymized, individual and trial-level data (analysis data sets), as well as other information (e.g., protocols and Clinical Study Reports), as long as the trials are not part of an ongoing or planned regulatory submission. This includes requests for clinical trial data for unlicensed products and indications. These clinical trial data can be requested by any qualified researchers who engage in rigorous, independent scientific research and will be provided following review and approval of a research proposal and Statistical Analysis Plan (SAP) and execution of a Data Sharing Agreement (DSA). Data requests can be submitted at any time and the data will be accessible for 12 months, with possible extensions considered. For more information on the process, or to submit a request, visit the following link: https://www.abbvie.com/our-science/clinical-trials/clinical-trials-data-and-information-sharing/data-and-information-sharing-with-qualified-researchers.html.

## Abbreviations

+: Positive
–: Negative
AE: Adverse event
ALK: Anaplastic lymphoma kinase
C: Cycle
C_max_: Maximum observed concentration
CNS: Central nervous system
CR: Complete response
D: Day
DOR: Duration of response
ECOG: Eastern cooperative oncology group
EGFR: Epidermal growth factor receptor
HNSCC: Head and neck squamous cell carcinoma
IHC: Immunohistochemistry
IP-10: Interferon gamma-induced protein 10
iRECIST: Immune response evaluation criteria in solid tumors
IV: Intravenous
KM: Kaplan–Meier
mAb: Monoclonal antibody
MedDRA: Medical dictionary for regulatory activities
MIG: Monokine induced by gamma interferon
NE: Not estimable
NSCLC: Non-small cell lung cancer
ORR: Objective response rate
PD: Pharmacodynamic
PD-1: Programmed cell death protein 1
PD-L1: Programmed cell death protein 1 ligand 1
PD-L2: Programmed cell death protein 1 ligand 2
PFS: Progression-free survival
PK: Pharmacokinetic
PR: Partial response
Q: Every
RECIST: Response evaluation criteria in solid tumors
SD: Stable disease
TEAE: Treatment-emergent adverse event
TRAE: Treatment-related adverse event
v: Version
w: Weeks

## Declarations

### Informed consents

This study was approved by the institutional review board at each participating site prior to initiation of any screening or study-specific procedures. The study was conducted in accordance with the Declaration of Helsinki and Good Clinical Practice guidelines, as defined by the International Conference on Harmonization. Written informed consent was obtained from each individual participating in the study.
