# Supplementary material for: First-in-human phase 1 study of budigalimab, an anti-PD-1 inhibitor, in patients with non-small cell lung cancer and head and neck squamous cell carcinoma
Source: Cancer Immunol Immunother. 2021 Jul 3;71(2):417–31. doi: 10.1007/s00262-021-02973-w (PMC8783908; doi:10.1007/s00262-021-02973-w)
Supplement: Supplementary file 1 — Supplementary file1 (PDF 727 KB) [file 262_2021_2973_MOESM1_ESM.pdf]

Cancer Immunology, Immunotherapy (submitted in 2020) – Antoine Italiano et al.

Supplementary Materials

**Supplementary Table 1. Study procedures: toxicity criteria for permanent discontinuation of budigalimab following a treatment-emergent adverse event**
Grade ≥2 drug-related uveitis, eye pain, or blurred vision that does not respond to topical therapy or improve to grade 1 severity
Grade ≥3 drug-related bronchospasm, hypersensitivity reaction, or IRR regardless of duration
Grade ≥3 drug-related thrombocytopenia lasting >14 days or associated with bleeding
Liver function tests meeting the following criteria<br>• AST or ALT >5× ULN for >14 days<br>• AST or ALT >10× ULN regardless of duration<br>• Total bilirubin >3× ULN<br>• Concurrent AST or ALT >3× ULN and total bilirubin >2× ULN
Grade ≥3 non-skin, drug-related AE lasting >14 days
Hemolysis of any grade (with the exception of hemolytic uremic syndrome and autoimmune hemolytic anemia managed per ASCO guidelines for management of immune-related AEs following treatment with immune checkpoint inhibitor therapy; Brahmer et al, 2018[a])
Grade 4 drug-related AEs or laboratory abnormalities with the exception of<br>• Grade 4 neutropenia<br>• Grade 4 lymphopenia or leukopenia<br>• Isolated grade 4 electrolyte abnormalities without clinical sequelae corrected within 72 hours
Dosing interruption >28 days from the next scheduled dose with the exception of<br>• Dosing interruptions to allow for prolonged steroid tapers to manage drug-related AEs<br>• Dosing interruptions >28 days that occur for non–drug-related reasons may be allowed if approved
Any AE, laboratory abnormality, or intercurrent illness that presents a substantial clinical risk to the patient with continued ABBV-181 treatment
[a]Brahmer JR, Lacchetti C, Schneider BJ, Atkins MB, Brassil KJ, Caterino JM, Chau I, Ernstoff MS, Gardner JM, Ginex P, Hallmeyer S, Chakrabarty JH, Leighl NB, Mammen JS, McDermott DF, Naing A, Nastoupil LJ, Phillips T, Porter LD, Puzanov I, Reichner CA, Santomasso BD, Seigel C, Spira A, Suarez-Almazor ME, Wang Y, Weber JS, Wolchok JD, Thompson JA, National Comprehensive Cancer Network . (2018) Management of immune-related adverse events in patients treated with immune checkpoint inhibitor therapy: American Society of Clinical Oncology Clinical Practice Guideline. J Clin Oncol 36(17):1714–1768. https://doi.org/10.1200/JCO.2017.77.6385<br>AE, adverse event; ALT, alanine aminotransferase; ASCO, American Society of Clinical Oncology; AST, aspartate aminotransferase; IRR, infusion-related reaction; ULN, upper limit of normal.

Supplementary Table 2. Patient disposition		
**Disposition**	**HNSCC (N=41)**[a,b]	**NSCLC (N=40)**[a,b]
**Median duration of exposure to budigalimab, days (range)**	72 (1–617)	71 (1–490)
**Number of Q2W doses of budigalimab, n (%)**[c]		
1 dose	1 (3)	1 (5)
2 doses	2 (6)	1 (5)
3 doses	2 (6)	3 (16)
4 doses	8 (26)	2 (11)
5–10 doses	10 (32)	6 (32)
10–20 doses	3 (10)	1 (5)
>20 doses	5 (16)	5 (26)
**Number of Q4W doses of budigalimab, n (%)**[b]		
1 dose	1 (10)	4 (19)
2 doses	2 (20)	6 (29)
3 doses	0	1 (5)
4 doses	2 (20)	5 (24)
5–10 doses	4 (40)	2 (10)
10–20 doses	1 (10)	3 (14)
>20 doses	0	0
**Budigalimab dose interruption, n (%)**		
No interruption	31 (76)	27 (68)
1 interruption	9 (22)	13 (33)
2 interruptions	1 (2)	0
**Budigalimab discontinuation, n (%)**	39 (95)	36 (90)
Adverse event	3 (7)	7 (18)
Progressive disease	36 (88)	28 (70)
Withdrawal by patient	0	1 (3)
**Study discontinuation, n (%)**	39 (95)	37 (93)
Adverse event	2 (5)	3 (8)
Progressive disease	37 (90)	24 (60)
Withdrawal by patient	0	6 (15)
Other	0	4 (10)

[a]HNSCC cohort: N=31; NSCLC cohort: N=19; [b]HNSCC cohort: N=10; NSCLC cohort: N=21. [c]The following adverse events were reported that led to dose interruption (note, patients could experience >1 adverse event): for HNSCC patients, 2 (4.9%) patients each with acute kidney injury, dyspnea; 1 (2.4%) patient each with abnormal general physical condition, adrenal insufficiency, bronchospasm, decreased appetite, device occlusion, diarrhea, dysphagia, fatigue, hypokalemia, infected neoplasm, large intestine obstruction, lung infection, mouth hemorrhage, nausea, pneumonia, pyrexia, and upper respiratory tract infection. For NSCLC patients, 2 (5%) patients each with hypercalcemia, upper respiratory tract infection; 1 (2.5%) patient each with acute kidney injury, anemia, diarrhea, disturbance in attention, femoral neck fracture, gait disturbance, hypothyroidism, influenza, infusion-related reaction, malignant neoplasm progression, microscopic colitis, pneumonia, pyrexia, transient ischemic attack.
HNSCC, head and neck squamous cell carcinoma; NSCLC, non-small cell lung cancer; Q, every; W, weeks.

**Supplementary Table 3. Summary of TRAEs of any grade occurring in ≥10% of patients**				
**By MedDRA preferred term, n (%)**	**HNSCC N=41**		**NSCLC N=40**	
	Any grade, n (%)	Grade ≥3, n (%)	Any grade, n (%)	Grade ≥3, n (%)
Any AE	26 (63)	4 (10)	23 (58)	5 (13)
Hypothyroidism	8 (20)	0	6 (15)	0
Diarrhea	6 (15)	1 (2)	2 (5)	0
Fatigue	2 (5)	0	5 (13)	0
Pruritus	6 (15)	0	3 (8)	0

AE, adverse event; HNSCC, head and neck squamous cell carcinoma; MedDRA, Medical Dictionary for Regulatory Activities; NSCLC, non-small cell lung cancer; TRAE, treatment-related AE.

Supplementary Table 4. TEAEs considered immune-mediated reactions				
**By MedDRA preferred term, n (%)**	**All Grades**		**Grade ≥3**	
	**HNSCC (N=41)**	**NSCLC (N=40)**	**HNSCC (N=41)**	**NSCLC (N=40)**
**Any AE, n (%)**	17 (42)	16 (40)	2 (5)	2 (5)
Hypothyroidism	7 (17)	6 (15)	0	0
Diarrhea	5 (12)	1 (3)	1 (2)	0
Pruritus	3 (7)	2 (5)	0	0
Adrenal insufficiency	1 (3)	0	0	0
Acute kidney injury	1 (2)	1 (3)	1 (2)	0
Dry skin	1 (2)	0	0	0
Generalized pruritus	1 (2)	0	0	0
Pneumonitis	1 (2)	1 (3)	0	0
Asthenia	0	1 (3)	0	0
Dyspnea	0	1 (3)	0	0
Fatigue	0	1 (3)	0	0
Genital pruritus	0	1 (3)	0	0
Hyperthyroidism	0	2 (5)	0	0
Hypophosphatemia	0	1 (3)	0	0
Immune-mediated hepatitis	0	1 (3)	0	1 (3)
Macular rash	0	1 (3)	0	0
Maculopapular rash	0	3 (8)	0	0
Microscopic colitis	0	1 (3)	0	1 (3)
Skin toxicity	0	1 (3)	0	0
Urticaria	0	1 (3)	0	0

AE, adverse event; HNSCC, head and neck squamous cell carcinoma; MedDRA, Medical Dictionary for Regulatory Activities; NSCLC, non-small cell lung cancer; TEAE, treatment-emergent AE.

Supplementary Table 5. TEAEs leading to budigalimab discontinuation				
By MedDRA preferred term, n (%)	Total TEAEs		Total TRAEs	
	HNSCC (N=41)	NSCLC (N=40)	HNSCC (N=41)	NSCLC (N=40)
**Any AE, n (%)**	7 (17)	10 (25)	2 (5)	3 (8)
Malignant neoplasm progression	2 (5)	3 (8)	0	0
Pneumonitis	1 (2)	1 (3)	1[a] (2)	1[a] (3)
Deterioration in general physical health	1 (2)	0	0	0
Hypothyroidism	1 (2)	1 (3)	1[b] (2)	1[b] (3)
Immune-mediated hepatitis	0	1 (3)	0	1[c] (3)
Acute kidney injury	0	1 (3)	0	1[b] (3)
Acute respiratory distress syndrome	1 (2)	0	0	0
Cardiac arrest	1 (2)	0	0	0
Confusion	0	1 (3)	0	0
Diarrhea	0	1 (3)	0	0
Disturbance in attention	0	1 (3)	0	0
Dyspnea	0	1 (3)	0	0
Intracranial hemorrhage	0	1 (3)	0	0
Neoplasm progression	1 (2)	0	0	0
Upper respiratory tract infection	0	1 (3)	0	0

[a]Grade 1. [b]Grade 2. [c]Grade 4.
AE, adverse event; HNSCC, head and neck squamous cell carcinoma; MedDRA, Medical Dictionary for Regulatory Activities; NSCLC, non-small cell lung cancer; TEAE, treatment-emergent AE; TRAE, treatment-related AE.

**Supplementary Table 6. Summary of TEAEs leading to budigalimab dose interruption**		
**By MedDRA preferred term, n (%)**	**HNSCC N=41**	**NSCLC N=40**
Any AE	14 (34)	15 (38)
Diarrhea	1 (2)	1 (3)
Fatigue	1 (2)	1 (3)
Acute kidney injury	2 (5)	1 (3)
Anemia	0	1 (3)
Decreased appetite	1 (2)	0
Pneumonia	1 (2)	1 (3)
Pyrexia	1 (2)	1 (3)
Upper respiratory tract infection	1 (2)	2 (5)
Dyspnea	2 (5)	0
Hypercalcemia	0	2 (5)
Infusion-related reaction	0	1 (3)
Malignant neoplasm progression	0	1 (3)
Thrombocytopenia	0	1 (2.5)
Adrenal insufficiency	1 (2)	0
Bronchospasm	1 (2)	0
Microscopic colitis	0	1 (3)
Device occlusion	1 (2)	0
Attention disturbance	0	1 (3)
Dysphagia	1 (2)	0
Femoral neck fracture	0	1 (3)
Gait disturbance	0	1 (3)
Abnormal general physical condition	1 (2)	0
Hypokalemia	1 (2)	0
Hypothyroidism	0	1 (3)
Infected neoplasm	1 (2)	0
Influenza	0	1 (3)
Large intestinal obstruction	1 (2)	0
Lung infection	1 (2)	0
Mouth hemorrhage	1 (2)	0
Nausea	1 (2)	0
Transient ischemic attack	0	1 (3)
AE, adverse event; HNSCC, head and neck squamous cell carcinoma; MedDRA, Medical Dictionary for Regulatory Activities; NSCLC, non-small cell lung cancer; TEAE, treatment-emergent AE.		

**Supplementary Table 7. Summary of grade 5 treatment-emergent adverse events[a]**		
**By MedDRA preferred term, n (%)**	**HNSCC N=41**	**NSCLC N=40**
Any AE[b]	8 (20)	7 (18)
Cardiac arrest	1 (2)	0
Upper respiratory tract infection	0	1 (3)
Abnormal general physical condition	1 (2)	0
Malignant neoplasm progression	4 (10)	6 (15)
Neoplasm progression	1 (2)	0
Acute respiratory distress syndrome	1 (2)	0
Dyspnea	0	1 (3)

[a]None considered related to budigalimab. [b]Some patients may have experienced more than 1 AE.

AE, adverse event; HNSCC, head and neck squamous cell carcinoma; MedDRA, Medical Dictionary for Regulatory Activities; NSCLC, non-small cell lung cancer.

Supplementary Table 8. Dose-normalized PK parameters		
**PK parameter (%)**	**250 mg Q2W (N=66)**	**500 mg Q4W (N=25)**
C_{max}	20.6 (36)	19.5 (27)
AUC_{inf}	208 (42)	222 (40)

AUC_{inf}, area under the curve from time 0 to infinity; C_{max}, maximum drug concentration; PK, pharmacokinetic; Q, every; W, weeks.

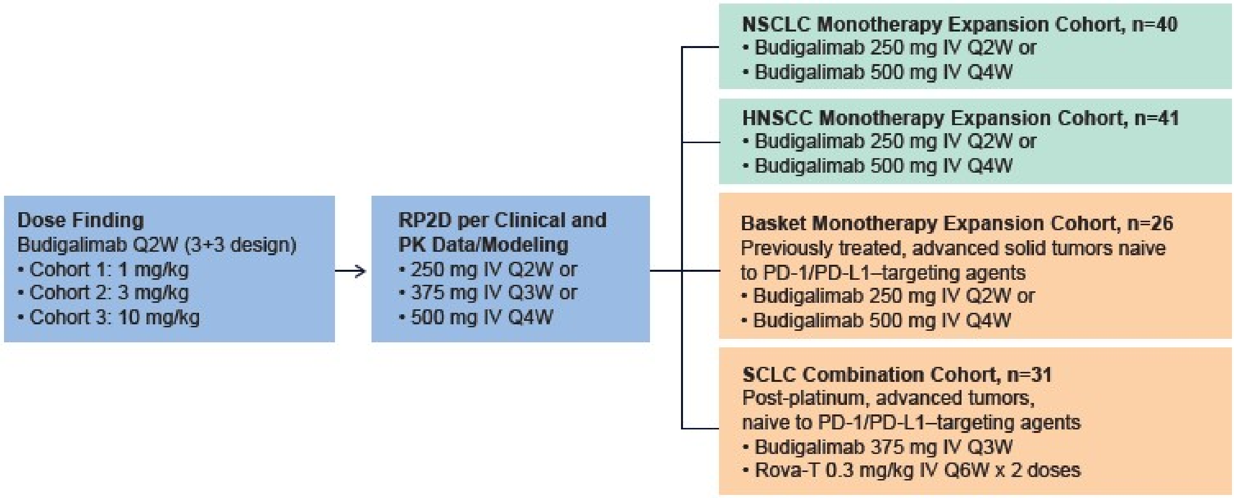

Supplementary Fig 1 Study schema. HNSCC, head and neck squamous cell carcinoma; IV, intravenous; NSCLC, non-small cell lung cancer; PD-1, programmed cell death protein 1; PD-L1, PD-1 ligand 1; PK, pharmacokinetic; Q, every; Rova-T, rovalpituzumab tesirine; RP2D, recommended phase 2 dose; SCLC small cell lung cancer; W, weeks

A

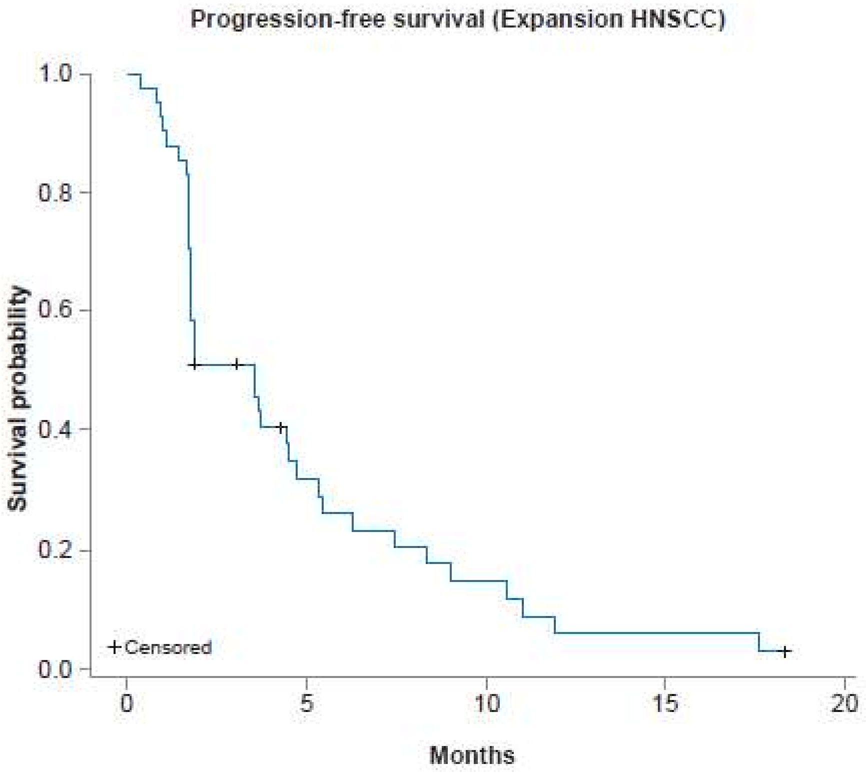

Progression-free survival (Expansion HNSCC)
Survival probability
1.0
0.8
0.6
0.4
0.2
0.0
+Censored
0
5
10
15
20
Months

B

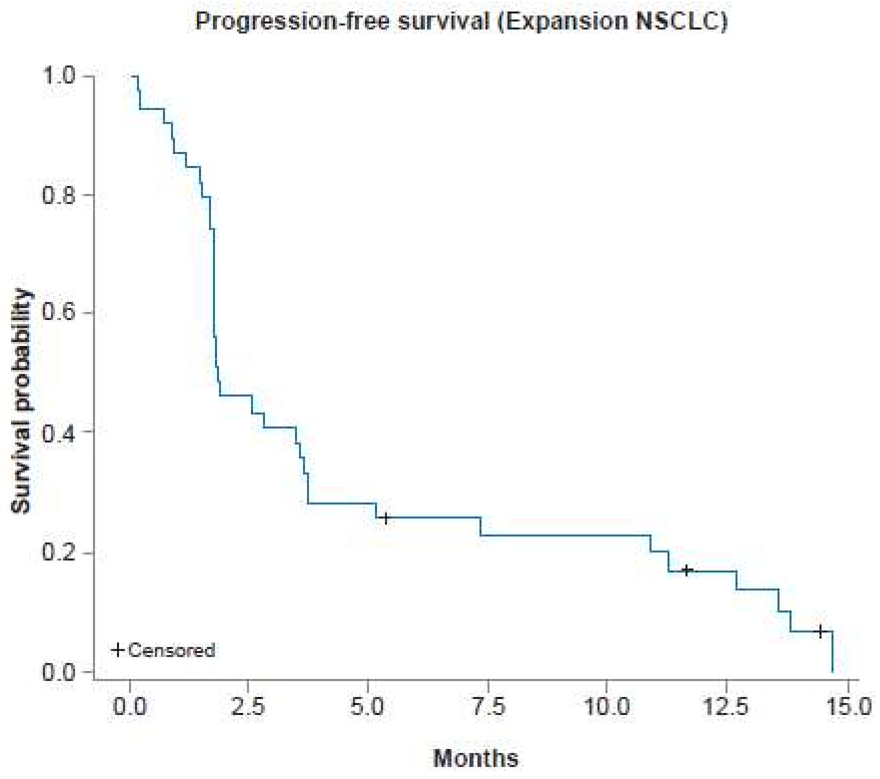

Supplementary Fig 2 Progression-free survival for A) HNSCC cohort and B) NSCLC cohort. HNSCC, head and neck squamous cell carcinoma; NSCLC, non-small cell lung cancer
